# Five Technologies for Detecting the *EGFR* T790M Mutation in the Circulating Cell-Free DNA of Patients With Non-small Cell Lung Cancer: A Comparison

**DOI:** 10.3389/fonc.2019.00631

**Published:** 2019-07-16

**Authors:** Yi-Lin Chen, Chien-Chung Lin, Shu-Ching Yang, Wan-Li Chen, Jian-Rong Chen, Yi-Hsin Hou, Cheng-Chan Lu, Nan-Haw Chow, Wu-Chou Su, Chung-Liang Ho

**Affiliations:** ^1^Molecular Diagnosis Laboratory, Department of Pathology, National Cheng Kung University Hospital, Tainan, Taiwan; ^2^Molecular Medicine Core Laboratory, Research Center of Clinical Medicine, National Cheng Kung University Hospital, Tainan, Taiwan; ^3^Association of Medical Technologists, Tainan, Taiwan; ^4^Department of Internal Medicine, National Cheng Kung University Hospital, Tainan, Taiwan; ^5^College of Medicine, Institute of Molecular Medicine, National Cheng Kung University, Tainan, Taiwan

**Keywords:** epidermal growth factor receptor, cell-free DNA, non-small cell lung cancer, amplification refractory mutation system, capillary electrophoresis, tyrosine kinase inhibitors

## Abstract

Third-generation tyrosine kinase inhibitors (TKIs) were developed to overcome T790M-mediated resistance to earlier generations of epidermal growth factor receptor *(EGFR)*-targeted TKIs. We compared four well-established and one in-house method for the analysis of the *EGFR* T790M mutation in plasma cell-free DNA (cfDNA), in hope to find a better way to select non-small cell lung cancer (NSCLC) patients appropriate for 3rd-generation TKI therapy. For sensitivity levels of each method, plasmid DNA with *EGFR* T790M mutations was serially diluted with cfDNA from healthy controls with wild type *EGFR*. The clinical performance was analyzed in a clinical cohort of *EGFR* mutation-positive NSCLC patients with acquired EGFR TKI resistance (*n* = 40). All methods except the therascreen kit (Qiagen) had a sensitivity level of 10 copies of T790M plasmid DNA in the spiked specimen. The detection rates of the *EGFR* T790M mutation in plasma cfDNA from the clinical cohort were 42.5, 35, 32.5, 22.5, and 17.5% for the in-house ARMS method, Bio-Rad droplet digital PCR, PANAMutyper, *Therascreen EGFR Plasma* RGQ PCR Kit and Cobas EGFR Mutation kit (with suboptimal template amounts), respectively. Osimertinib was given to 17 of 20 patients with *EGFR* T790M mutations. The best treatment responses, based on the RECIST criteria, included 6 partial responses (PR) and 7 stable diseases (SD). The PANAMutyper and the Bio-Rad droplet digital PCR were comparable, the Cobas EGFR Mutation kit required significantly more template for testing. The best combination would be the in-house ARMS method plus the PANAMutyper or Bio-Rad droplet digital PCR, which would have a detection rate of 50% (20/40) and a disease control rate of 76% (13/17).

## Introduction

The clinical outcomes of non-small cell lung cancer (NSCLC) patients whose tumors have activated mutations of epidermal growth factor receptor (*EGFR*) have improved since the introduction of *EGFR* tyrosine kinase inhibitors (*EGFR*-TKIs). However, drug resistance eventually appears for almost all patients treated with *EGFR*-TKIs. Secondary *EGFR*c.2369C>T (T790M) mutationsaccount for 30–50% of resistance to first- and second-generation TKIs (gefitinib, erlotinib, and afatinib) at about 9–12 months after the initial therapy (1, 2). The T790M mutation emerges when the cancer progresses (3); it is the putative gatekeeper residue (4). An irreversible third-generation TKI (osimertinib) (AZD9291) was developed to evade the bulky residue and efficaciously sensitized *EGFR* and T790M mutations. It led to progression-free survival for 7–10 months (5, 6). It also led to a substantial improvement of patients with metastatic NSCLC whose cancer progressed after first-generation TKI treatment and whose tumors had the T790M mutation (7, 8). However, osimertinib is toxic to healthy tissue that expresses wild-type (WT) EGFR, most notably the skin, gastrointestinal tract, and eyes [Drugs@FDA] (9). Therefore, a highly specific and sensitive method for detecting a secondary T790M mutation is needed to select an appropriate treatment regimen for NSCLC patients with good disease control.

Analysis of genetic alterations in tumor tissue during drug resistance is always difficult for patients with poor disease control. Other situations that make repeated biopsies impossible include inaccessible tumors and tumors with high vascularity or an air bronchogram. Detecting genetic mutations in the cell-free DNA (cfDNA) extracted from patient plasma in a non-invasive liquid biopsy has been used as a surrogate for the molecular evolution of tumor tissue (10). Many technologies have been developed to increase the sensitivity of detecting genetic mutations in cfDNA. Amplicon-based studies have shown that cfDNA is highly fragmented and most commonly exists in ~100–150 base pairs (bp) (11, 12). There are several kits with analytical sensitivities between 1 and 3%: Therascreen (Qiagen Manchester Ltd, Manchester, UK), Cobas (*Roche* Molecular Systems *Inc*., South Branchburg, NJ, USA), and PANAMutyper™ R EGFR (Panagene, Daejeon, Korea), and a <1% sensitivity droplet digital polymerase chain reaction (ddPCR) (Bio-Rad Laboratories Inc., Hercules, CA, USA).

The T790M mutation site is in exon 20 in a GC-rich region. Moreover, there is a single nucleotide polymorphism (SNP) (silent Q787Q) only eight nucleotides upstream of T790M, which challenges the design of molecular testing (13). Because cfDNA is highly fragmented, primers and probes must contain a short GC-rich sequence and avoid the potential interference of Q787Q. This leaves little room for complicated experimental designs. Therefore, we used a straightforward amplified refractory mutation system (ARMS) strategy which combined a high melting temperature and high-resolution *capillary* electrophoresis (QIAxcel Advanced System high-resolution capillary electrophoresis; Qiagen GmbH, Hilden, Germany to increase the specificity and sensitivity of detecting T790M in cfDNA. We evaluated the performance of 5 different platforms for detecting *EGFR* T790M in cfDNA and the subsequent treatment responses in *EGFR* mutation-positive NSCLC patients with acquired TKI resistance.

## Materials and Methods

### Patients

Between January 2015 and January 2016, National Cheng Kung University Hospital (NCKUH) analyzed the T790M cfDNA in 10 mL of peripheral blood from each of 40 NSCLC patients with *EGFR*-sensitizing mutation and acquired EGFR-TKI resistance. All patients signed a written informed consent form for molecular analysis. Recorded demographic and clinical data included age, sex, tumor staging, Eastern Cooperative Oncology Group Performance Status (ECOG PS), treatment regimens, and maximal response. This study was approved by the NCKUH Institutional Review Board (IRB #: A-BR-101-129 and B-ER-106-082).

### Treatment Response Evaluation

The response to osimertinib (Tagrisso™) was assessed using Response Evaluation Criteria in Solid Tumors (RECIST) guidelines 1.1.

### Cell-Free DNA Extraction

The peripheral blood was transferred at ambient temperature to our laboratory within 60 min after blood drawing. Cell-free DNA (cfDNA) was isolated from 1 mL of plasma using a kit (QIAamp Circulating Nucleic Acid kit; Qiagen Manchester Ltd, Manchester, UK) according to the manufacturer's instructions. The yield of cfDNA was quantified by a Qubit fluorometer (Thermo Fisher, Waltham, MA). To ensure the absence of genomic DNA contamination, 2 μl cfDNA was dilutedin sterilewaterto a final volume of 15 μl and submitted to capillary electrophoresis using QiaxcelScreenGel according to manufacturer's instructions. Only those without high-molecular weight DNA were used for diagnoses.

The targets and control primers for the in-house method were:

**Table:** 

**Primer**	**Sequence (5′>3′)**	**Conc.** **(μM)**
T790M-forward	CCTCCACCGTGCAHCTCATCAT	10
T790M-reverse	CCGTATCTCCCTTCCCTGATT	10
Internal control-forward *TBXAS1*/X9U	GCCCGACATTCTGCAAGTCC	2.5
Internal control-reverse *TBXAS1*/X9R	CCACAACGGCCCTTCCCAA	2.5

The test was done in duplicate with 21 μL of Super Therm Gold Master Mix (Bionovas Biotechnology, Toronto, Ontario, Canada), 2 μL of T790M primers (10 μM), 2 μL of control primers (2.5 μM), and 2 μL of working DNA (15 ng/μL) in a final volume of 25 μL. The PCR assay was done under the following conditions: at 95°C (5 min), 35 cycles of 95°C (30 s), 63°C (30 s), and 72°C (30 s) and then at 73°C for (20 s). The primers of the human *TBXAS1* gene (exons 9; GenBank accession no. D34621) produced 100-bp amplified products to indicate the quality of the PCR reaction (14). The expected PCR product sizes for T790M and the control allele (*TBXAS1/*X9) were 150 and 100 bp, respectively.

### Reference Real-Time PCR Platforms for Comparison

#### Roche Cobas EGFR Mutation Test v2

The Cobas EGFR Mutation Test v2 can identify 42 mutations in exons 18-21: L858R, exon 19 deletions, L861Q, and TKI-resistant mutation T790M. It was designed to test tissue and plasma specimens and mixed-batch tissue and plasma on the same plate. Additionally, a cfDNA sample preparation kit was developed and optimized for extracting DNA from plasma. In addition, a semi-quantitative index (SQI) was included in the report to reflect a trend in the amount of mutant cfDNA during tumor progression.

#### Qiagen Therascreen EGFR Plasma RGQ PCR Kit

The *therascreen* EGFR Plasma RGQ PCR Kit was designed to detect *EGFR* exon 19 deletions, exon 20 T790M and exon 21 L858R, and to provide a qualitative assessment of the mutation status. The test kit combines ARMS PCR and Scorpion primers to improve the sensitivity and specificity of detecting single-base mutations. All procedures used the manufacturer's instructions.

#### PANAMutyper™ R EGFR Kit

The assay was designed to detect 47 different *EGFR* variants based on peptide nucleic acid (PNA)-mediated real-time PCR clamping and melting-peak analysis. PNA is used to construct the PCR clamp reactions, in which the clamp suppresses the amplification of WT DNA and increases the preferential amplification of mutant sequences. The PCR assay was done under the following conditions: (1) for two holding periods at 50°C for 2 min and 95°C for 15 min; (2) 15 cycles at 95°C for 30 s, at 70°C for 20 s, and at 63°C for 60 s; and (3) 35 cycles at 95°C for 10 s, at 53°C for 20 s, and at 73°C for 20 s. A melting-curve step was performed at 95°C for 15 min, at 35°C for 5 min, and at 35–75°C with temperature increments of 0.5°C for 3 s to acquire fluorescence values on all four channels (FAM, HEX, ROX, and Cy5). The melting peaks were derived from melting-curve data. The mutant-type DNA-specific detection probe with fluorescent dye and quencher was genotyped using melting-peak analysis.

#### Droplet Digital PCR

A digital PCR system (QX200 Droplet Digital™ PCR [ddPCR] System; Bio-Rad) was developed to optimize the qPCR assay. The ddPCR mixture contained 8 μL of 2 × ddPCRSupermix, 400 nM of primers for both albumin and T790M, and 125 nM of probe. The entire 20 μL reaction was loaded into a droplet cartridge (Bio-Rad) according to protocol and the cartridge placed in the droplet generator (Bio-Rad#186-3002). Then, a vacuum was applied to the cartridge to draw the PCR reagents and oil through a flow-focusing nozzle where around 20,000 uniform nanoliter-sized droplets were formed and suspended in an emulsion (15).

The emulsion was transferred into a 96-well plate (Eppendorf, Hamburg, Germany) and sealed using a foil lid and thermal plate sealer (Eppendorf). The sealed plates were cycled using a C-1000 thermal cycler (Bio-Rad) under the following conditions: 10 min hold at 95°C, 40 cycles at 94°C for 30 s, and then at 55°C for 60 s. After they had been amplified, the PCR-positive and -negative droplets were separately counted in the droplet reader by passing them in a single stream through a fluorescence detector. PCR-positive droplets, which contain at least one copy of the targets, are more fluorescent than are PCR-negative droplets. The number of PCR-positive and -negative droplets for each fluorophore (e.g., FAM and HEX) in the samples was measured using QuantaSoft™ (Bio-Rad). The fraction of the PCR-positive droplets was then fitted to a Poisson distribution to determine the absolute starting copy number in units of copies/μL input samples. The criterion for PCR-positive mutant alleles was set at ≥3 mutants within 300 droplets.

### Sensitivity Levels of Different Methods for Detecting *EGFR* T790M Mutations

A vector plasmid (pGEM-T Easy Vector; Promega, Madison, WI, USA) with T790M mutations was derived from human primary NSCLC tissue using PCR cloning. The sequence of the plasmid was confirmed using Sanger sequencing. To evaluate the sensitivity levels of each method, plasmid DNA with *EGFR* T790M mutations was serially diluted with cfDNA from healthy controls with WT *EGFR*. The sensitivity levels of *EGFR* T790M detection were verified using diluent containing 10^4^, 10^3^, 10^2^, 10, and at least 1 copy of plasmid DNA. Each condition was repeated at least three times.

## Results

### Patient Characteristics

The study cohort included 20 women and 20 men (mean age: 60 years; age range: 45–83 years) (Table 1). One patient had stage II NSCLC, but the other 39 patients had stages IIIa to IV. Thirty-nine patients (97.5%) had adenocarcinoma and one patient (2.5%) had thyroid transcription factor-1 (TTF-1) negative NSCLC. Thirty patients (75%) were non-smokers. One patient had the G719X *EGFR* mutation, 17 had exon 19 deletion, one had exon 20 insertion, and 21 had the L858R *EGFR* mutation. All patients were treated with first-line TKIs (gefitinib in 24; erlotinib in 7; afatinib in 9), and acquired resistance to EGFR-TKIs developed within 1.5 years.

**Table 1:** Characteristics of *EGFR* mutation-positive NSCLC patients with acquired resistance to EGFR-TKIs (*n* = 40).

	**No. of patients (%)**	**Detection of T790M mutation in plasma (%)**
Total	40 (100)	20 (50.0)
Median age (range) in years	60 (45–83)	
**GENDER**		
Male	20 (50.0)	11 (55.0)
Female	20 (50.0)	9 (45.0)
**SMOKING HISTORY**		
Never-smoker	30 (75.0)	14 (70.0)
Smoker	10 (25.0)	6 (30.0)
***EGFR*** **MUTATION STATUS AT DIAGNOSIS**		
Exon 19 deletion	17(42.5)	10 (50.0)
L858R or L858P	21 (52.5)	9 (45.0)
Other	2 (5.0)	1 (5.0)
**TUMOR STAGE**		
I–II	1 (2.5)	1 (5.0)
III	6 (15.0)	3 (15.0)
IV	33 (82.5)	16 (80.0)
First EGFR-TKI		
Gefitinib	24 (60.0)	11 (55.0)
Erlotinib	7 (17.5)	4 (20.0)
Afatinib	9 (22.5)	5 (25.0)

### Using Cloned *EGFR* DNA Fragments to Determine Sensitivity Levels of Different Assays

**Figure 1:**
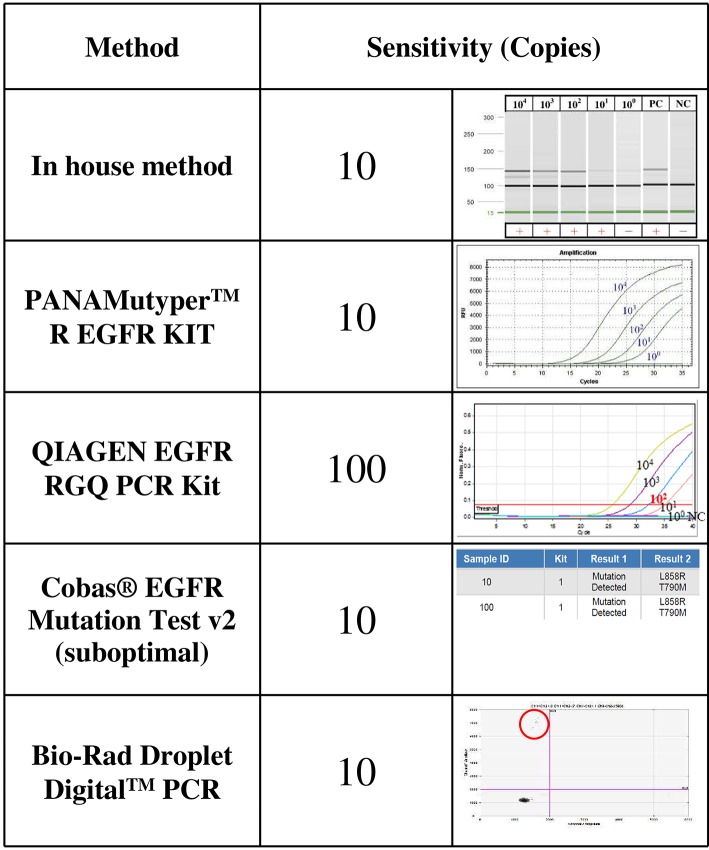
The sensitivity levels of *EGFR* T790M mutation detection using different assays. The T790M mutation was cloned and serially diluted in 30 ng/μL of cfDNA from healthy controls. One microliter of diluent that contained 10^4^, 10^3^, 10^2^, 10^1^, or about 1 copy of T790M plasmid DNA was analyzed three times using each detection method.

The sensitivity levels of different methods were compared using the same spiked plasmid DNA (with a 235-bp insert covering T790M) prepared in the laboratory. All but the therascreen kit (Qiagen) detected 10 copies of T790M plasmid DNA mixed with cfDNA from healthy controls; the *therascreen*kit, however, detected 100 copies (Figure 1).

### Comparison of *EGFR* T790M Mutation Detection Rates in Patients' Plasma

**Figure 2:**
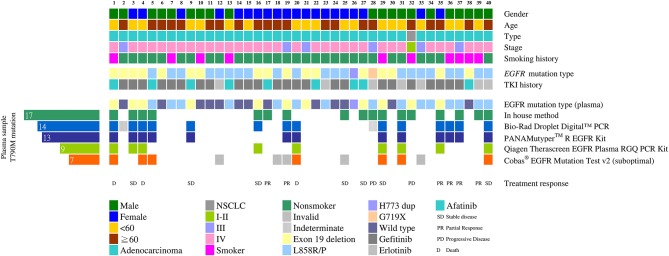
Detecting the *EGFR* T790M mutation using different cfDNA assay methods (*n* = 40). Clinical characteristics of the 40 patients are indicated by different colors on the top five rows. The middle two rows show the initial mutation status of the tumor tissue and the TKI used after the initial diagnoses. The initial tissue samples were tested using the *therascreen* method. Mutation patterns in the cfDNA detected using different methods are shown in the heat map at the bottom. The number of *EGFR* T790M mutation-positive plasma samples is shown on the left.

The plasma T790M mutation was detected in 17 patients (42.5%) using the in-house method, in 14 (35%) patients using the Bio-Rad ddPCR method, in 13 (32.5%) patients using the PANAMutyper method, in 9 (22.5%) patients using the *therascreen* method, and in 7 (17.5%) patients using the Cobas method. The cumulative detection rate for the T790M mutation was 50% (Figure 2).

The Cobas method was used most frequently under suboptimal conditions in this study. When we used 50 ng of sample DNA as recommended by the protocol, there might not be enough DNA left for the other four methods. In 35 cases we used <50 ng DNA (range: 25.0–49.5 ng) for the Cobas and 5 tests yielded invalid results. In addition, the Bio-Rad digital PCR method yielded two indeterminate results: for these 2 patients, only100 droplets instead of the required 300 droplets were obtained. The test conditions were all satisfactory for the *in-house*, PANAMutyper and *therascreen* assays.

### Clinical Responses to EGFR-TKIs

**Figure 3:**
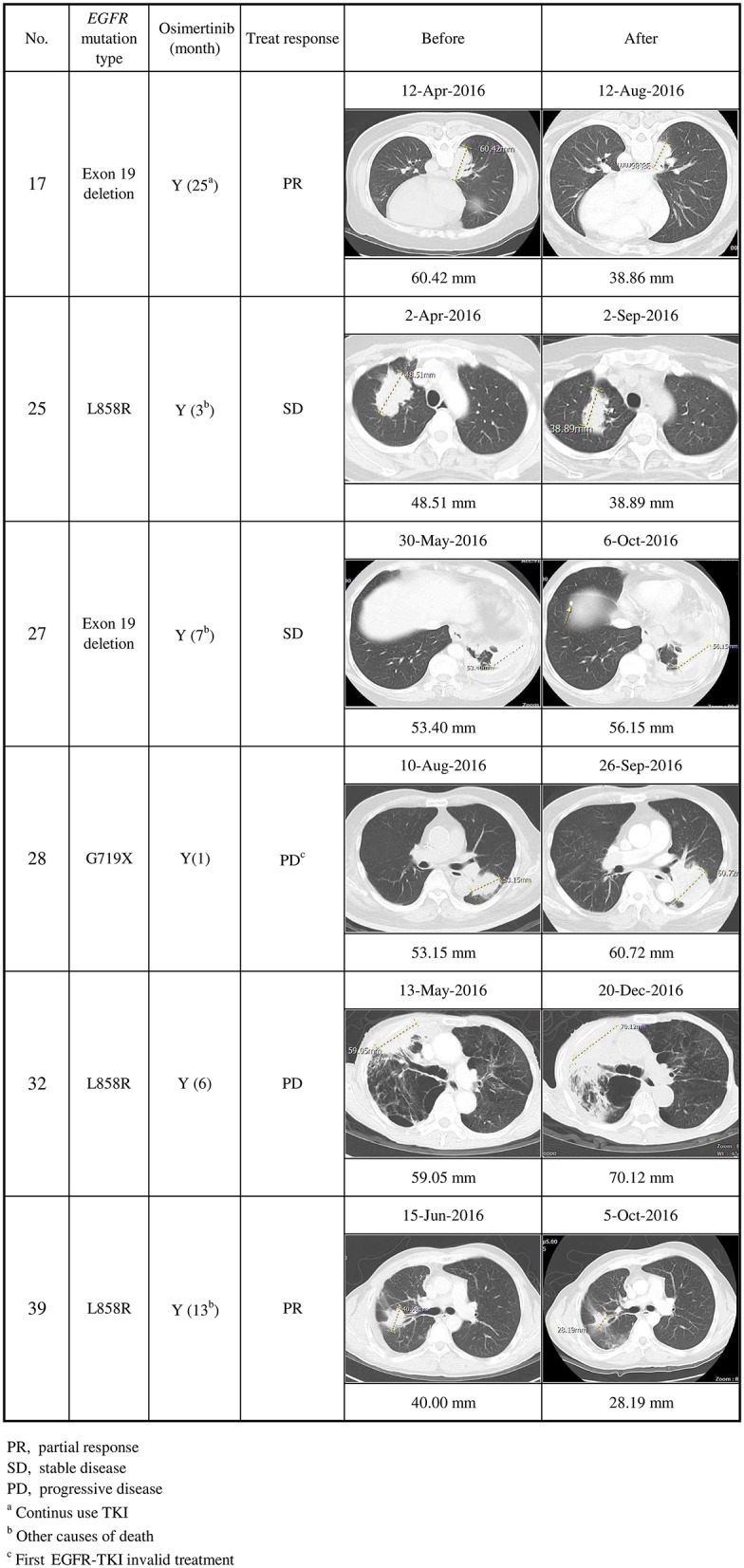
Clinical characteristics of secondary *EGFR* T790M mutations detected using the in-house method only, and their responses to osimertinib treatment (*n* = 6). Cases 17 and 39 partially responded, cases 25 and 27 stabilized, and cases 28 and 32 progressed after osimertinib treatment.

Seventeen of the 20 *EGFR* T790M mutation-positive patients detected using at least one method of plasma DNA testing were subsequently treated with osimertinib. Thirteen of them (76%) stabilized or partially responded, as defined in the RECIST criteria. Six patients (cases 17, 25, 27, 28, 32, and 39) were detected using the in-house method only (Figures 2, 3): cases 25 and 27 stabilized, cases 17 and 39 partially responded, and cases 28 and 32 progressed after osimertinib treatment (Figure 3). Case 28 had an uncommon *EGFR* G719X mutation when initially diagnosed and showed inferior response to erlotinib for 3 months. The tumor was also resistant to osimertinib treatment, which was stopped after 1 month. Case 32 had an *EGFR* L858R mutation that was initially treated with gefitinib from December 2015 to May 2016. After the cancer had progressed and the *EGFR* T790M mutation was detected in plasma, the tumor was treated with osimertinib for 6 months, but it grew from 59.05 to 72 mm.

**Figure 4:**
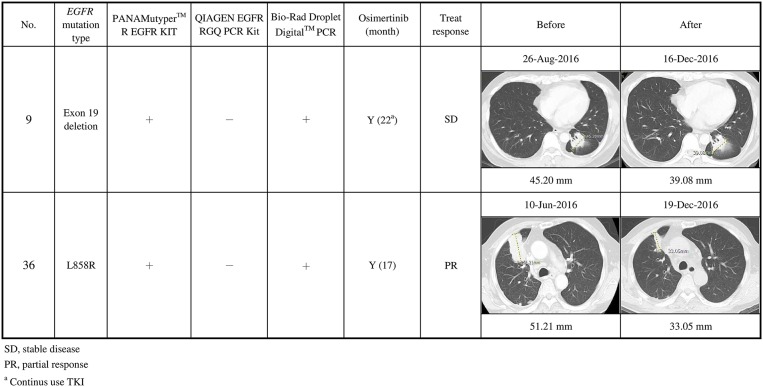
Clinical characteristics of secondary *EGFR* T790M mutations not detected using the in-house method, and their responses to osimertinib treatment (*n* = 2). Case 9 stabilized, and case 36 partially responded after osimertinib treatment.

In contrast, cases 9, 20, and 36 were detected using Bio-Rad ddPCR and PANAMutyper, but not using the in-house method. Case 9 stabilized and case 36 partially responded (Figure 4). Patient 20 died before being treated with osimertinib.

Of the 13 cases that had stabilized or partially responded, 2 had not been detected using the in-house method, while 4 were not detected using Bio-Rad ddPCR or PANAMutyper. Combining the in-house method with either the Bio-Rad ddPCR or PANAMutyper method would have detected all of them.

## Discussion

Secondary *EGFR* T790M mutation accounts for 30–50% of resistance to the earlier-generation TKIs about 9–12 months after the initial treatment (1, 2). We found that, in a cohort of 40 *EGFR* mutation-positive NSCLC patients with acquired EGFR TKI resistance, the cumulative detection rate for the T790M mutation in cfDNA from all five methods was 50% (*n* = 20): 3 had died before osimertinib therapy, and 13 had stabilized or partially responded after osimertinib therapy. Had only a single detection method been used, the detection rate would have been lower, and patients who might have benefited from osimertinib therapy would have been missed. However, because we combined the in-house method with the Bio-Rad ddPCR or the PANAMutyper method, our detection rate was 50%. The in-house method is relatively inexpensive; thus, the combination of two methods is financially feasible for routine practice.

Detecting the T790M mutation in plasma cfDNA is becoming more popular and soon will most likely be the preferred method for deciding whether to recommend osimertinib therapy. One study reported that of their 91 T790M mutation patients, 85 were detected using plasma cfDNA and the other 6 plasma-negative patients were detected using a tumor re-biopsy (16). Eighty-nine of them were then treated with osimertinib; the response rate was 72% (64/89), and the disease control rate (CR + PR + SD) was 83% (74/89). Our study had a disease control rate of 76% (13/17), probably because our sample size was smaller and less homogeneous.

Detecting the *EGFR* T790M mutation in cfDNA requires a sensitive method. Different commercial kits claim various sensitivities, but direct comparison of their performance is difficult to find in the literature. We did not intend to determine the detection limit of each method. Instead, we used a spiked sample across different methods to stratify them into different groups of sensitivity levels. We found that the *therascreen EGFR Plasma* RGQ PCR kit (Qiagen) was the least sensitive method with a sensitivity level of 100 instead of 10 copies. Accordingly, it detected only 9 of the 20 positive cases. We do not recommend it.

It is very difficult to design a sensitive test for short fragmented DNA with high GC content, such as *EGFR* T790M in cfDNA. All commercial kits try to design sensitive T790M tests with short amplicons for cfDNA, ranging from 80 bp (BioRad ddPCR) to 118 bp (Roche Cobas® EGFR mutation test). However, when amplicons are short, the high GC content would impose design constraints which negatively affect the sensitivity of the T790M test, especially for complicated designs such as a long Scorpion probe/primer. That may be the reason why the Qiagen system suffers the most in the T790M test. The amplicon is 150 bp long for our in-house method. It is on the long end of cfDNA. The length allowed us to avoid the high GC area for primer designs, leading to 6 more positive cases detected by the in-house method only. However, it also missed 3 cases, probably because other methods are able to detect shorter, more fragmented cfDNA which cannot be detected by the in-house method, resulting in the observed false negativity.

One of the limitations of this study was the absence of tissue biopsy as a reference for a systematic comparison. For example, Castellanos-Rizaldos et al. used tissue biopsy as a gold standard and validated a sensitive allele-specific qPCR test that overcomes the limited abundance of the mutation by simultaneously capturing and interrogating exosomal RNA/DNA and cfDNA (exoNA) (17). In addition to the added input from exosomal RNA/DNA, the workflow included a pre-amplification step using a wild-type blocker to enrich the mutant amplicons. With the boosted mutant input, the mutant-specific qPCR achieved 92% sensitivity and 89% specificity comparing to tumor biopsy results. This provided another way to tackle the design challenge of EGFR T790M mutation tests. For the 40 cases in this study, only 6 of them were clinically suitable and personally willing to take the risk of tissue biopsy. All 6 showed consistent results between cfDNA and tissue biopsy (4 positive; 2 negative).

It is known that cfDNA shows dynamic kinetics in plasma (18). The sensitivity of circulating tumor DNA detection was substantially improved using serial sampling in one breast cancer study (19). In our study, however, cfDNA T790M mutation analysis was done as a single time-point assay. We used the in-house ARMS tests in duplicate to eliminate possible sampling bias caused by the low copy number of target templates. Indeed, 3 of the 17 positive results (cases 5, 16, and 37) detected using the in-house ARMS were because of the duplicate assays.

A liquid biopsy normally uses fresh samples. There are additional concerns using formalin-fixed paraffin-embedded (FFPE) tissue samples to detect *EGFR* T790M mutations. It is well-known that deamination of nucleotides in FFPE samples causes the C:G > T:A change, which, in turn, yields a false-positive T790M C > T mutation result (20). To prevent this, uracil DNA glycosylase (UDG), which cleaves deaminated cytosine (uracil), has been included in some commercial kits. In our protocol for FFPE samples, UDG (5 U/μL) was added to a reaction mixture that contained 30 ng of FFPE-derived DNA. We recommend analyzing paired samples with and without UDG when detecting *EGFR* T790M mutations in FFPE samples. If possible, it is desirable to analyze the non-tumorous parts using the same fixation and embedding processes to gauge the background deamination artifact.

In summary, we found that a high-cost commercially available kit combined with our low-cost in-house ARMS method yielded a 50% detection rate of the *EGFR* T790M mutation in plasma cfDNA from 40 patients with secondary resistance to earlier generation EGFR TKIs. A large prospective cohort study is required to evaluate the clinical significance of detecting this mutation in the plasma cfDNA of NSCLC patients.

## Data Availability

The raw data supporting the conclusions of this manuscript will be made available by the authors, without undue reservation, to any qualified researcher.

## Author Contributions

Y-LC and C-LH conceived and designed the experiments. S-CY, W-LC, J-RC, and Y-HH performed the experiments. Che-CL analyzed the data. Chi-CL and W-CS contributed reagents, materials, analysis tools. N-HC, Y-LC, and C-LH wrote the paper.

### Conflict of Interest Statement

The authors declare that the research was conducted in the absence of any commercial or financial relationships that could be construed as a potential conflict of interest.
